# Prospective observational study of 2 wearable strain sensors for measuring the respiratory rate

**DOI:** 10.1097/MD.0000000000038818

**Published:** 2024-07-19

**Authors:** Hiroki Sato, Tatsuya Nagano, Shintaro Izumi, Jun Yamada, Daisuke Hazama, Naoko Katsurada, Masatsugu Yamamoto, Motoko Tachihara, Yoshihiro Nishimura, Kazuyuki Kobayashi

**Affiliations:** a Division of Respiratory Medicine, Department of Internal Medicine, Kobe University Graduate School of Medicine, Hyogo, Japan; b Graduate School of System Informatics, Kobe University, Hyogo, Japan; c Department of Respiratory Medicine, Kitaharima Medical Center, Hyogo, Japan.

**Keywords:** respiratory rate, strain sensor, vital signs, wearable sensor

## Abstract

The respiratory rate is an important factor for assessing patient status and detecting changes in the severity of illness. Real-time determination of the respiratory rate will enable early responses to changes in the patient condition. Several methods of wearable devices have enabled remote respiratory rate monitoring. However, gaps persist in large-scale validation, patient-specific calibration, standardization and their usefulness in clinical practice has not been fully elucidated. The aim of this study was to evaluate the accuracy of 2 wearable stretch sensors, C-STRECH® which is used in clinical practice and a novel stretchable capacitor in measuring the respiratory rate. The respiratory rate of 20 healthy subjects was measured by a spirometer with the stretch sensor applied to 1 of 5 locations (umbilicus, lateral abdomen, epigastrium, lateral chest, or chest) of their body at rest while they were in a sitting or supine position before or after exercise. The sensors detected the largest amplitudes at the epigastrium and umbilicus compared to other sites of measurement for the sitting and supine positions, respectively. At rest, the respiratory rate of the sensors had an error of 0.06 to 2.39 breaths/minute, whereas after exercise, an error of 1.57 to 3.72 breaths/minute was observed compared to the spirometer. The sensors were able to detect the respiratory rate of healthy volunteers in the sitting and supine positions, but there was a need for improvement in detection after exercise.

## 1. Introduction

The respiratory rate is a vital indicator of patients’ general condition, and it can be used to detect early-stage changes in the severity of illness.^[1]^ An abnormal respiratory rate (tachypnea, hypopnea) is associated with poor prognosis and in-hospital death.^[2]^ However, the respiratory rate is measured in approximately 30% of hospitalized patients because the standard method relies on visual counts, which are time-consuming. Moreover, its importance is not recognized by medical staff.^[3]^ If an appropriate monitoring device was developed, the physical condition of the patient could be evaluated in real-time and in a simple manner, and a prompt response to changes in the patients’ condition could be expected.

Indeed, several methods of measuring the respiratory rate, such as monitoring respiratory airflow, chest wall movements and modulation of cardiac activity, are used in real-time, but the utility of these methods varies depending on the type of measurement.^[4–6]^ Some methods are more prone to motion artifacts, which makes them difficult to use during exercise, and other machines are too large to carry or wear on the body. For example, impedance-based respiratory rate monitoring using electrocardiography electrodes, which is currently widely used in clinical practice, is easy to use but prior reports show them to be imprecise with limits of agreement of −9.9 to 7.5 breaths/minutes.^[7]^ Moreover, electrodes have difficulty adhering to the body surface when the patient is sweating or may cause irritation of the skin after a prolonged period of use, and they are susceptible to body movement, which can cause motion artifacts.^[8]^

The wearable strain sensor measures respiratory waveforms by being stretched, which changes its capacitance in accordance with the expansion and contraction of the chest or abdomen during breathing.^[9]^ The sensor is small, lightweight, and suitable for monitoring patients. However, their performance has not been fully clarified.

The aim of this study was to evaluate the accuracy and function of 2 wearable stretch sensors, C-STRECH® (Bando Chemical Industries, Hyogo, Japan) which is commercially available as a respiratory sensor and a novel stretchable capacitor (TOYOBO, Osaka, Japan) in measuring the respiratory rate.

We evaluated which part of the body has the highest amplitude of respiratory waveforms and is most suitable for wearing the sensors. Moreover, we aimed to evaluate the accuracy of the sensors compared to that of spirometry and the effect of exercise on the measurements.

## 2. Methods

### 2.1. Design

This was a prospective observational study performed at Kobe University Hospital. Volunteers who were at least 18 years of age at the time of consent, able to exercise for 3 to 5 minutes at a rate of 7 Mets (150 watts on a bicycle ergometer) to 10 Mets (approximately 161 to 200 watts on a bicycle ergometer) and who could provide written consent for the study were considered eligible for this study. Participants were enrolled from May 2023 to July 2023. Ethical approval for this study was provided by the Kobe University Hospital Clinical and Translational Research Center ethics committee (permission number: B220190) on February 6, 2023. The study conformed to the Declaration of Helsinki and Good Clinical Practice guidelines. A written informed consent was obtained from all participants.

### 2.2. Measurement of the respiratory rate

**Figure 1.**
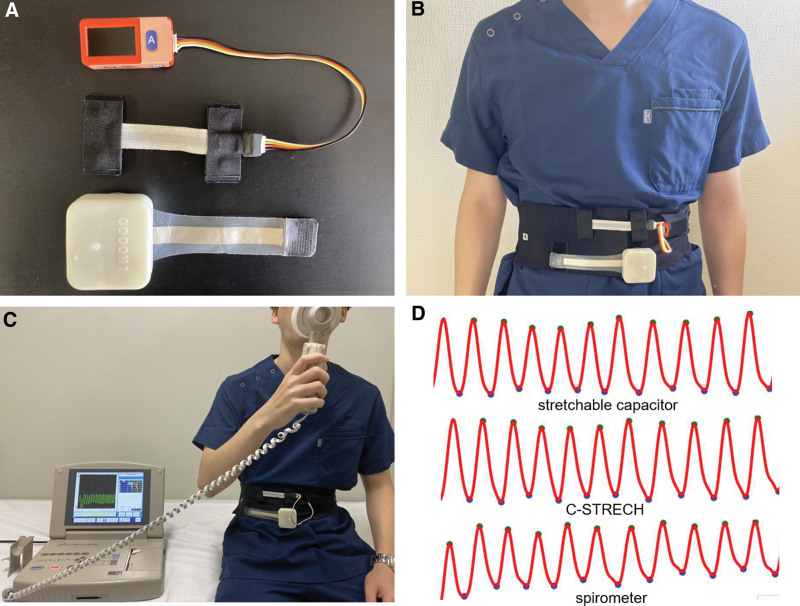
The devices, participants and the waveform obtained from measurements. (A) The stretchable capacitor and the C-STRECH® sensor. (B) The participant with the sensors worn around the body. On the lower side, the stretchable capacitor was used, and on the upper side, C-STRECH® was attached for measurement. (C) Participants in the sitting position. While the measurements were performed, the participants were breathing through a spirometer. (D) Representative waveforms of each sensor were obtained from participants during rest at the umbilicus.

During the pre-observation period, the age, sex, height, weight, blood pressure, heart rate (pulse rate), respiratory rate, body temperature and oxygen saturation measured by pulse oximetry (SpO_2_) were measured. We used 2 sensors, C-STRECH® (Bando Chemical Industries, Hyogo, Japan) and a stretchable capacitor (TOYOBO CO., Osaka, Japan) (Fig. 1A). The participants wore a flexible band around their abdomen or chest to monitor their respiratory rate with 2 strain sensors attached while they were breathing at rest or after exercise (Fig. 1B). The band had hook and loop tapes to attach the C-STRECH® in the upper part and a stretchable capacitor in the lower part. The mechanism of the sensors is described in detail elsewhere.^[9]^ Each sensor is made of an elastomer and electrode layers, which constitute a parallel plate structure that enables it to function as a capacitor. The capacitance of the sensor is linearly related to the strain of the area of interest so that the sensors can detect the expansion of the body site to which it is attached. These materials are compact in size and lightweight, which makes them suitable for portable use. The recorded data from these devices were transferred wirelessly to the researchers’ tablets or personal computers. Measurements were obtained at 5 points, including the umbilicus, lateral abdomen, epigastrium, center of the chest, and lateral chest, with the patient sitting and in the supine position. One minute was taken for recording each position, and the actual respiratory rate was monitored via spirometry (HI-801, CHEST CO., Tokyo, Japan) (Fig. 1C). Additionally, 20 seconds of breath holding was recorded while the patients were in the sitting and supine positions at the umbilicus.

The time shifts between devices were manually corrected to match the measurement period of the spirometer. No incomplete waveforms were removed. Additionally, a digital high-pass filter with a cutoff frequency of 0.1Hz was applied to the initial measurement waveform to remove baseline fluctuations.

The amplitude was measured for each respiratory cycle. Then average amplitude for each position and sensors were calculated for each participant. The average values were calculated for the same interval as the spirometer. The average amplitude of the waveform recorded on the umbilicus with the sensor of interest was used as a control to calculate the relative amplitude for sitting and supine positions respectively (Fig. 1D). The relative amplitude for each site, body position and sensor type was calculated as (the target average amplitude)/(average amplitude of the umbilicus). The average amplitude for each sensor at the umbilicus for each participant was considered to be the reference for themselves. Therefore, we set the relative amplitude for umbilicus as 1.0 in each participant considering the sensor of interest. After the resting measurements had finished, the participants exercised with an ergometer (V67i, Senoh Corporation, Chiba, Japan) at 150 W for 3 minutes. The respiratory rate in the sitting position and supine position was recorded at the umbilicus of the participants. Post-exercise blood pressure, heart rate, respiratory rate, body temperature, SpO_2_, modified Borg scale score and adverse events were recorded for each participant.

### 2.3. Outcomes

The main outcomes were the peak heights of the waveforms at rest in the sitting or supine position with the sensors attached at 5 locations: the umbilicus, lateral abdomen, epigastrium, chest and lateral chest. The accuracy of the sensors was evaluated by the mean absolute differences between spirometrically detected breathing and a stretchable capacitor or C-STRECH^®^ detected breathing at rest and post-exercise. Finally, the rate of adverse events was recorded.

### 2.4. Statistical analysis

The data are expressed as the median (range) or mean (95% CI). We used Kruskal–Wallis tests with Bonferroni correction to compare the relative amplitudes between the different body sites and Wilcoxon signed rank test to compare vital signs. The statistical significance level was set to *P* < .05. The data were analyzed using Python 3.10 and EZR software (Saitama Medical Center, Jichi Medical University, Saitama, Japan), a graphical user interface for R (The R Foundation for Statistical Computing, Vienna, Austria) and a customized version of the R commander.^[10]^

## 3. Results

### 3.1. Subject characteristics

A total of 20 volunteers were included in the study. Fourteen (70%) patients were male, the median age at enrollment was 34 years (range, 25–48), the average height was 169.5 cm (range, 152–180), and the average body weight was 63 kg (range, 45–92), with a body mass index (BMI) of 22.6 kg/m^2^ (range, 18.9–31.8) (Table 1).

**Table 1:** Descriptive characteristics of the participants.

Variables		n = 20	
Male	(%)	14	(70)
Age (yr)	[median (range)]	34	(25–48)
height (cm)	[median (range)]	169.5	(152–180)
weight (kg)	[median (range)]	63	(45–92)
BMI (kg/m²)	[median (range)]	22.6	(18.9–31.8)

BMI = body mass index.

### 3.2. Differences in the amplitude of waveforms considering the monitoring position

**Figure 2.**
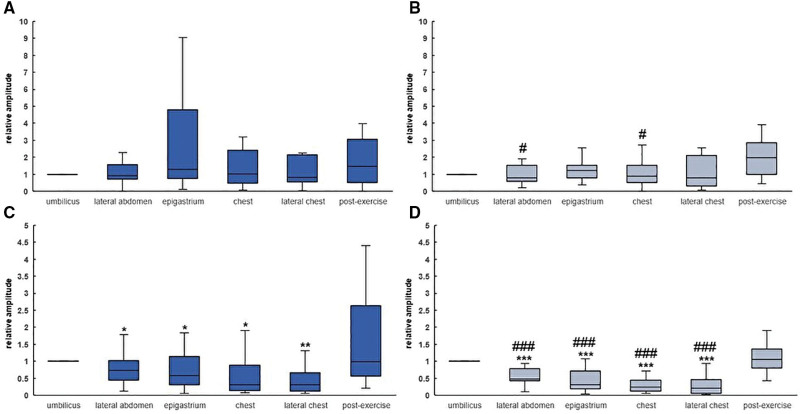
Amplitude in sitting and supine positions. The amplitude at each site was compared to the amplitude measured at the umbilicus while the patient was sitting in the supine position (n = 18). The figures show (A) stretchable capacitor at sitting and (B) C-STRECH® while sitting. Amplitudes in the supine position representative of the (C) stretchable capacitor, (D) C-STRECH®. *P < .05; **P < .01; ***P < .001 for differences from the umbilicus. #*P* < .05; ###*P* < .001 for differences from post-exercise.

In the supine position, the epigastrium had the largest amplitude for the both stretchable capacitor (1.30; range, 0.11–66.5) and C-STRECH® (1.24; range, 0.38–3.04) (Fig. 2). In the supine position, the stretchable capacitor and C-STRECH® had the largest amplitude in the umbilicus, which was significantly greater than that at the other sites of measurement. The results for men and women were similar for the site with the largest median (Supplemental Fig. S1, http://links.lww.com/MD/N122, S2, http://links.lww.com/MD/N123). After exercise, the amplitudes in the sitting and supine positions were 1.46 (range, 0.21–42.1) and 0.99 (range, 0.01–31.1) for the stretchable capacitor and 1.96 (range, 0.43–2.99) and 1.05 (range, 0.43–7.01) for the C-STRECH® device, respectively. There was no significant difference between amplitude at the umbilicus before and after exercise.

### 3.3. The accuracy of respiratory monitoring for assessing the respiratory rate

The average respiration intervals were calculated from the measurement data of 2 sensors and spirometry for each measurement condition, except for breath-hold. A discrete Fourier transform was performed on the recorded waveforms of each sensor, and the average respiratory interval in seconds was calculated from the frequency peaks.

Table 2 shows the results of the calculated error of each sensor relative to the spirometer. The results for Subject 1 and Subject 3 are excluded from this calculation due to unstable measurement results. The differences in the respiratory rate obtained from spirometry and calculated from the stretchable capacitor or C-STRETCH® device in the sitting position were greatest in the chest for both sensors, with 1.11 breaths/min (95% CI [−0.32, 2.55]) and 1.17 breaths/min (95% CI [−0.37, 2.70]), respectively. In the supine position, the differences were greatest in the epigastrium, at 2.39 breaths/min (95% CI [−0.39, 5.17]) and 0.89 breaths/min (95% CI [−0.40, 2.18]). In contrast to men, whose results were similar to those of the total population, among women in the sitting position, the differences were greatest in the lateral abdomen and not in the chest (Supplemental Table. 1, http://links.lww.com/MD/N120, 2, http://links.lww.com/MD/N121). The sensor-detected respiration rate post-exercise was 1.57 to 3.72 breaths/minute greater than that of the spirometer, which tended to be greater than that at rest, depending on the position and type of sensor.

**Table 2:** The mean absolute difference in the respiratory rate of the 2 sensors considering the site and position of sensing.

Sensing site	Position	Stretchable capacitor	95% CI	C-strech	95% CI
		Mean absolute difference		Mean absolute difference	
		(times/min)		(times/min)	
Umbilicus	Sitting	0.06	(−0.06, 0.17)	0.11	(−0.05, 0.27)
	Supine	0.06	(−0.06, 0.17)	0.11	(−0.05, 0.27)
Lateral abdomen	Sitting	0.17	(−0.02, 0.36)	0.22	(−0.14, 0.59)
	Supine	0.06	(−0.06, 0.17)	0.06	(−0.06, 0.17)
Epigastrium	Sitting	0.56	(−0.50, 1.61)	0.61	(−0.44, 1.66)
	Supine	2.39	(−0.39, 5.17)	0.89	(−0.40, 2.18)
Chest	Sitting	1.11	(−0.32, 2.55)	1.17	(−0.37, 2.70)
	Supine	0.06	(−0.06, 0.17)	0.06	(−0.06, 0.17)
Lateral chest	Sitting	0.17	(−0.02, 0.36)	0.11	(−0.05, 0.27)
	Supine	0.06	(0.17, −0.17)	0.11	(−0.05, 0.27)
Post-exercise	Sitting	3.72	(0.56, 6.88)	2.61	(−0.40, 5.62)
	Supine	1.89	(−1.02, 4.80)	1.57	(−1.34, 4.49)

CI = confidence interval.

### 3.4. Safety and adverse events

All participants were able to finish the exercise and recording procedure. The vital signs of the participants before and after the procedure are shown in Table 3. The median modified Borg scale was 4.5 (range, 2–7), which indicated moderate effort of the participants, and the respiratory rate determined by the observer and heart rate were significantly elevated compared to those before exercise, with no significant change in SpO_2_. No adverse events were reported during or after the study.

**Table 3:** Changes in the vital signs and measurements before exercise.

Measurements	Timing of measurement		n = 20	
		Median	Range	*P* value
Body temperature (°C)	Pre-exercise	36.6	(35.8–37.2)	.97
	Post-exercise	36.7	(36.1–37.3)	
Systolic blood pressure (mm Hg)	Pre-exercise	120.5	(106–139)	.53
	Post-exercise	120.5	(100–138)	
Diastolic blood pressure (mm Hg)	Pre-exercise	76.5	(60–90)	.67
	Post-exercise	75.5	(59–98)	
Heart rate (beats/min)	Pre-exercise	75.5	(61–103)	<.001
	Post-exercise	95.5	(77–120)	
Respiratory rate (times/min)	Pre-exercise	12	(12–20)	<.001
	Post-exercise	21	(12–34)	
SpO_2_ (%)	Pre-exercise	97	(95–99)	.23
	Post-exercise	97	(95–98)	
Modified Borg scale	Post-exercise	4.5	(2–7)	N/A

SpO2 = oxygen saturation measured by pulse oximetry.

## 4. Discussion

In this prospective observational study, we found that stretch sensors attached to the body had the largest amplitude in the epigastrium while sitting and the umbilicus while in the supine position, which was consistent between the 2 sensors. This result suggested that, in the investigated areas in this study, the site that had the largest movement differed according to the position of the participant. The movement in the epigastrium was greater than that in the umbilicus in the sitting position due to compression of the abdomen.

Differences between the respiratory monitoring and spirometry results were <1 time/minute except for at the site for which the largest difference occurred. The site that had the largest difference changed from the chest to the epigastrium after the participants moved their bodies to the supine position, suggesting that some sites of measurements may be avoided in certain body positions. Discrepancies in the site with the most errors according to sex could be because the chest area may be difficult to fit with the device due to undergarments worn in daily life and differences in body shape, such as breast morphology, between men and women. Finally, there was a greater difference in the number of measurements after exercise than at rest.

To date, several methods have been used to monitor breathing.^[11–13]^ Consistent with our findings, prior studies have reported that stretch sensors such as inductive or capacitive sensors are able to accurately measure the respiratory rate.^[9,14]^ For example, a study using a capacitor reported an error of 0.01 ± 1.90 time/minute while sitting or standing.^[15]^ Comparing the results with other methods measuring respiratory rate considering the site of measurement, 1 study attaching an accelometer at the clavicle, chest or abdomen had an error smaller than ± 2 breaths/min.^[16]^ Photoplethysmography had an error of 0.6 to 3 breaths/min at rest while measuring several parts of the body including fingers, arms and the forehead.^[17]^ Another study evaluating the accuracy of a stretch sensor in measuring respiratory rate demonstrated differences between the site of measurement such as umbilicus, upper abdomen, xiphoid process and upper thorax with the largest error of 1.05 to −0.90 breaths/min in the upper abdomen.^[18]^

A spirometer measures the airflow caused by breathing and is considered to be an accurate and reliable method of measuring respiration, with few artifacts caused by motion.^[19]^ However, wearing it on the body or carrying around is not suitable, as is wearing a wearable device, and there is discomfort due to the need to continuously apply the mouthpiece during measurement. Wearable sensors are thought to improve these problems while maintaining measurement accuracy. The stretch sensors manufactured by Bando Chemical and Toyobo are identical in principle, are considered to be functionally equivalent, and are portable wireless devices.

Our study involved wearing bands around the subject body, which has several merits. It can be used without removing or changing clothes, and the discomfort is less because it does not contact the skin. Moreover, it can be attached when the subject has a wet surface when sweating occurs, and the band can be reused and carried and used in various situations.

The strength of this study is that we measured chest wall movements at multiple sites and at multiple positions with 2 sensors. We were able to record the differences in thoracic and abdominal movements between the sitting and supine positions. This approach can lead to better knowledge of the best site for monitoring the respiratory rate when using methods that record the movement of the torso.

Two different stretch sensors were simultaneously attached to the same site. One of the sensors, C-STRECH® has been studied in measuring of respiratory rate during 6-minute walking test in patients with or without chronic obstructive pulmonary disease and during exercise.^[9,20]^ Consistent results were shown between the sensors considering the amplitude of the respiratory waveforms and differences from spirometry-recorded breathing. Therefore, our study suggests that the novel capacitor produced by TOYOBO® has a similar accuracy compared to C-STRECH® in counting respiratory rate and measuring the movement of the chest or abdomen.

This study has 2 main limitations. First, the study size was small, the population was composed of a healthy, young, homogeneous group of volunteers, and 2 patients were excluded. Participant 1 was excluded due to recording errors due to mishandling by the researcher, and participant 3 had sensing errors because of their BMI, which made it difficult for the bands to attach to the body. We have shown that the sensors are able to track the respiratory rate in this population, except for 2 participants excluded from the analysis. Nevertheless, further studies with a larger population that includes more elderly individuals or subjects who have respiratory illness and who have abnormal chest or abdominal movement during respiration should be conducted. The second limitation is that the sensor-detected respiratory rate under exercise load conditions was 1.57 to 3.72 breaths/minute larger than that detected via spirometry. This may be due to an increase in the respiratory rate or an increase in abdominal movement resulting from the use of respiratory accessory muscles such as abdominal muscle groups. In clinical use, it is important to detect abnormalities in the respiratory rate, including tachypnea, at an early stage, and improvement is necessary in this respect.

## 5. Conclusions

Our analysis suggested that the 2 stretch sensors in our study are able to record chest and abdominal movement during respiration and the respiratory rate of healthy volunteers in the sitting and supine positions, with the need for improvement after exercise.

## Acknowledgments

The authors thank the volunteers for participating in this study and TOYOBO Co., Ltd., and Bando Chemical Industries Ltd., for providing the stretchable strain sensor.

## Author contributions

**Conceptualization:** Hiroki Sato, Tatsuya Nagano.

**Data curation:** Hiroki Sato.

**Formal analysis:** Hiroki Sato.

**Funding acquisition:** Tatsuya Nagano.

**Investigation:** Hiroki Sato, Shintaro Izumi, Jun Yamada.

**Methodology:** Tatsuya Nagano, Shintaro Izumi, Masatsugu Yamamoto.

**Project administration:** Yoshihiro Nishimura.

**Supervision:** Shintaro Izumi, Daisuke Hazama, Motoko Tachihara, Yoshihiro Nishimura, Kazuyuki Kobayashi.

**Writing – original draft:** Hiroki Sato.

**Writing – review & editing:** Hiroki Sato, Tatsuya Nagano, Shintaro Izumi, Jun Yamada, Daisuke Hazama, Naoko Katsurada, Masatsugu Yamamoto, Motoko Tachihara, Yoshihiro Nishimura, Kazuyuki Kobayashi.

## Supplementary Material








