# Supplementary figures and images for: Prospective observational study of 2 wearable strain sensors for measuring the respiratory rate
Source: Medicine (Baltimore). 2024 Jul 19;103(29):e38818. doi: 10.1097/MD.0000000000038818 (PMC11398755; doi:10.1097/MD.0000000000038818)

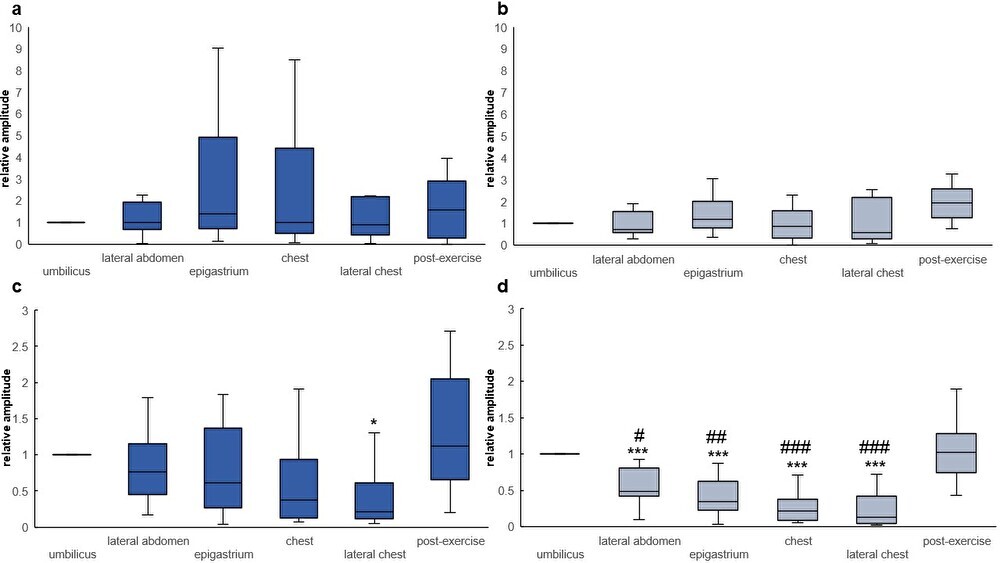

Supplement: Supplementary file 1 [file medi-103-e38818-s001.jpg]

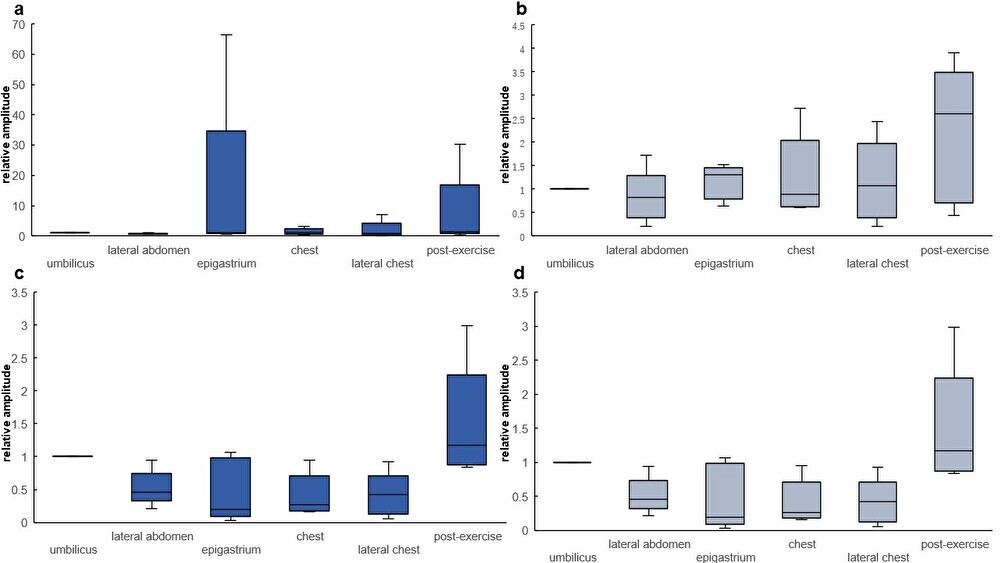

Supplement: Supplementary file 2 [file medi-103-e38818-s002.jpg]
