# Supplementary material for: Prospective observational study of 2 wearable strain sensors for measuring the respiratory rate
Source: Medicine (Baltimore). 2024 Jul 19;103(29):e38818. doi: 10.1097/MD.0000000000038818 (PMC11398755; doi:10.1097/MD.0000000000038818)
Supplement: Supplementary file 3 [file medi-103-e38818-s003.docx]

| **Supplementary Table S1 The mean absolute difference in respiratory rate of men (n=13)** | | | | | |
| --- | --- | --- | --- | --- | --- |
|  |  | **STRECHABLE CAPACITOR** |  | **C-STRECH** |  |
| **Sensing site** | **Position** | **mean absolute**  **difference** | **95% CI** | **mean absolute**  **difference** | **95% CI** |
|  |  | **(times/minute)** |  | **(times/minute)** |  |
| umbilicus | sitting | 0.00 | (-0.17, 0.17) | 0.14 | (-0.09, 0.37) |
|  | supine | 0.07 | (-0.10, 0.24) | 0.14 | (-0.09, 0.37) |
| lateral abdomen | sitting | 0.14 | (-0.13, 0.41) | 0.07 | (-0.10, 0.24) |
|  | supine | 0.00 | (-0.17, 0.17) | 0.00 | (-0.17, 0.17) |
| epigasitrium | sitting | 0.71 | (-0.81, 2.24) | 0.71 | (-0.81, 2.23) |
|  | supine | 2.43 | (-1.45, 6.30) | 0.57 | (-0.79, 1.93) |
| chest | sitting | 1.36 | (-0.69, 3.41) | 2.00 | (-0.20, 4.20) |
|  | supine | 0.07 | (-0.10, 0.24) | 0.07 | (-0.10, 0.24) |
| lateral chest | sitting | 0.21 | (-0.06, 0.48) | 0.14 | (-0.09, 0.37) |
|  | supine | 0.07 | (-0.10, 0.24) | 0.21 | (-0.02, 0.45) |
| postexcercise | sitting | 4.86 | (0.46, 9.26) | 3.64 | (-0.63, 7.91) |
|  | supine | 4.07 | (-0.14, 8.28) | 3.73 | (-0.49, 7.96) |
| Abbreviations: CI, confidence interval | | | | | |
