# Supplementary material for: Prospective observational study of 2 wearable strain sensors for measuring the respiratory rate
Source: Medicine (Baltimore). 2024 Jul 19;103(29):e38818. doi: 10.1097/MD.0000000000038818 (PMC11398755; doi:10.1097/MD.0000000000038818)
Supplement: Supplementary file 4 [file medi-103-e38818-s004.docx]

| **Supplementary Table S2 The mean absolute difference in respiratory rate of women (n=5)** | | | | | |
| --- | --- | --- | --- | --- | --- |
|  |  | **STRECHABLE**  **CAPACITOR** |  | **C-STRECH** |  |
| **Sensing site** | **Position** | **mean absolute**  **difference** | **95% CI** | **mean absolute**  **difference** | **95% CI** |
|  |  | **(times/minute)** |  | **(times/minute)** |  |
| umbilicus | sitting | 0.20 | (-0.36, 0.76) | 0.00 | (0.00, 0.00) |
|  | supine | 0.00 | (0.00, 0.00) | 0.00 | (0.00, 0.00) |
| lateral abdomen | sitting | 0.20 | (-0.36, 0.76) | 0.60 | (-1.07, 2.27) |
|  | supine | 0.20 | (-0.36, 0.76) | 0.20 | (-0.36, 0.76) |
| epigasitrium | sitting | 0.20 | (0.00, 0.00) | 0.20 | (-0.36, 0.76) |
|  | supine | 1.80 | (-2.54, 6.14) | 1.60 | (-2.84, 6.04) |
| chest | sitting | 0.20 | (-0.36, 0.76) | 0.20 | (-0.36, 0.76) |
|  | supine | 0.00 | (0.00, 0.00) | 0.00 | (0.00, 0.00) |
| lateral chest | sitting | 0.00 | (0.00, 0.00) | 0.00 | (0.00, 0.00) |
|  | supine | 0.00 | (0.00, 0.00) | 0.00 | (0.00, 0.00) |
| postexcercise | sitting | 3.00 | (-4.65, 10.65) | 2.61 | (-4.97, 10.57) |
|  | supine | 0.40 | (-0.28, 1.08) | 1.57 | (-0.36, 0.76) |
| Abbreviations: CI, confidence interval | | | | | |
